# Effectiveness of Different Neuromuscular Recovery Strategies in Elite Youth Female Football Players

**DOI:** 10.3390/sports13020036

**Published:** 2025-01-29

**Authors:** Silvia Sedano, Sergio Maroto-Izquierdo

**Affiliations:** 1 Department of Health Sciences, European University Miguel de Cervantes,47012 Valladolid, Spain; smaroto@uemc.es; 2 i+HeALTH Strategic Research Group, Department of Health Sciences, European University Miguel de Cervantes, 47012 Valladolid, Spain; 3 Proporción A, Applied Sports Science Centre, 47015 Valladolid, Spain

**Keywords:** fatigue, wellness, stretching, foam roller, mobility, vertical jump, women

## Abstract

This study aimed to assess the effectiveness of various active recovery strategies in youth female soccer players during competitive tournaments with limited recovery periods (i.e., 24–48 h). Twenty-two elite under-17 female football players participated in this randomized controlled trial, which encompassed fourteen 90 min official matches. Participants were randomly allocated to one of three recovery protocols: passive stretching, foam rolling, or lumbopelvic mobility exercises, which were implemented ten minutes after each match. Countermovement jump with free arm (CMJA) height was measured pre-intervention, immediately post-intervention, and 5 h post-intervention. Wellness perception was evaluated 24 h later. Significant enhancements in CMJA height were observed immediately after all recovery protocols and at 5 h post-intervention compared with pre-intervention (*p* < 0.001). The lumbopelvic mobility protocol yielded the most substantial improvement, significantly surpassing both stretching and foam rolling. Moreover, significant increases in wellness perception were observed following the foam rolling (*p* < 0.001, ES = 0.95) and mobility (*p* < 0.05, ES = 0.88) protocols, with the mobility protocol demonstrating a marginally larger effect size than stretching. Active recovery strategies significantly enhanced neuromuscular function and wellness perception in under-17 female soccer players. Lumbopelvic mobility exercises exhibited superior efficacy, suggesting that they should be prioritized in post-match recovery regimens.

## 1. Introduction

Football is arguably the most popular team sport worldwide, and data from the UEFA indicate a significant increase in the number of female players in Europe over the past decade [1]. Given the high level of participation, frequency of competition, rising standards of professionalism, and demanding physical requirements, it is essential to implement multidisciplinary strategies [2]. These strategies should aim to optimize performance and health while also including the need to address specific recovery strategies for female players.

Football is a complex sport characterized by high-intensity activities that impose significant strain on neuromuscular parameters [3,4,5]. This leads to muscle soreness, temporary reductions in physical performance (such as explosive force generation), an increased risk of injury, and hindered post-match recovery [6,7,8,9,10]. Understanding physiological changes after a football match is essential for developing effective recovery strategies, especially during competitive tournaments with limited recovery times (i.e., 24–48 h) [6]. It is also crucial to enable players to perform at their best, as key performance indicators such as sprinting or jumping performance tend to decrease immediately after a match [10,11]. Furthermore, prolonged exposure to fatigue resulting from insufficient recovery time may increase the risk of injury rate in subsequent matches [12,13]. However, adopting recovery strategies, particularly those aimed at enhancing mobility and strength (i.e., knee flexors) plays a crucial role in mitigating the risk of injuries associated with high training loads and match intensity [13,14,15].

A variety of passive recovery strategies are widely adopted in sports, including football, such as rest and sleep, cold water immersion, massage, compression garments, and electrical stimulation [16,17,18]. However, active recovery is believed to enhance the recovery process by accelerating the return to homeostasis after a match, mitigating muscle fatigue and soreness, restoring energy levels and performance metrics, and allowing athletes to withstand higher training volumes, thereby improving their overall performance [19]. Consequently, several post-match (i.e., immediately after a match up to 72 h) active recovery techniques have been employed, including self-myofascial release [20], mobility and stability exercises [21], dynamic stretching [22], and low-intensity aerobic or resistance exercise training [6,23,24], to promote recovery and prevent injuries [15]. However, considerable debate in research studies complicates the ability to draw definitive conclusions and establish practical guidelines.

Although theoretical perspectives suggest that post-match active recovery strategies implemented immediately after competition enhance training load tolerance, research on their effects in female football players is limited, and the results are equivocal. Andersson et al. [6] reported that submaximal cycling and low-intensity resistance training performed after a match had no significant effects on the recovery of neuromuscular and biochemical parameters in female football players. However, the effects of other active recovery strategies performed immediately after a match, which have been demonstrated to be effective in other sports contexts, remain unknown in female football players. These strategies include joint mobility exercises and strength exercises that target the core muscles. Moreover, to the best of our knowledge, no studies have evaluated the effectiveness of an active recovery program performed between two consecutive matches in female football players [25] nor have they compared three active recovery strategies in the same sample. Additionally, while many current recovery approaches are adapted from research in male football [11,14,15,26,27,28,29,30], the potential physiological and biomechanical differences between male and female players underscore the need to tailor evidence-based methods to the specific needs of female athletes [31]. Such adaptations are crucial to ensure effective recovery and meet the growing demands of modern football.

Given the lack of scientific evidence regarding proper active intervention strategies to accelerate post-match recovery, this study aimed to evaluate the effectiveness of active recovery during competitive tournaments with limited recovery times (i.e., 24–48 h) in young female players. Additionally, this study aimed to compare three different recovery strategies: stretching, foam rolling, and lumbopelvic mobility protocols. We hypothesized that all three strategies would be effective in improving neuromuscular function, with the lumbopelvic mobility protocol obtaining the best results in this particular sample.

## 2. Materials and Methods

### 2.1. Study Design

This randomized controlled trial consisted of 14 90 min official football matches and two familiarization sessions. The first two sessions familiarized the participants with the assessment and recovery intervention protocols. Two different recovery strategies (foam rolling and lumbopelvic mobility exercises) were compared with traditional passive stretching. Ten minutes after each official match (pre), participants performed a countermovement jump with free arms (CMJA). They were then assigned to a 10 min recovery strategy program in a counterbalanced and randomized order. The CMJA height was assessed immediately after the recovery protocol (post) and 5 h later (post 5 h). In addition, wellness perception was assessed 24 h later.

### 2.2. Participants

Twenty-two elite under-17 female football players (16.2 ± 0.6 years, 62.0 ± 3.4 kg, and 166.0 ± 8.2 cm) voluntarily participated in this study. All of them belonged to the same team and played for at least 50 min in at least one of the 14 official matches included in this study. The participants’ training frequency was 4.1 ± 0.9 days/week with a mean duration for each session of 90 min. All matches included in this study were always played in tournaments over three days (two matches per tournament), with a recovery period of 24–48 h. The recovery interventions were randomized and counterbalanced across the participants to ensure a balanced and randomized fatigue protocol. Thus, each player was randomly assigned to one of the three recovery protocols (i.e., stretching, foam rolling, or lumbopelvic mobility) in a manner that ensured that an equal number of players experienced each protocol after a match, and across the different matches and recovery periods. This approach helped minimize any potential bias and ensured that the effects of each recovery strategy could be accurately assessed. Goalkeepers were excluded from this study because their training load and physical demands differed from those of field players. Similarly, players who sustained a musculoskeletal injury during the data collection period or within six months prior to this study were not included in this study. The players and their parents/tutors were fully informed about the experimental procedures before the intervention and provided informed consent to participate. This study was conducted in accordance with the Declaration of Helsinki and approved by the University Ethics Committee.

### 2.3. Procedures

#### 2.3.1. Recovery Protocols

**Figure 1:**
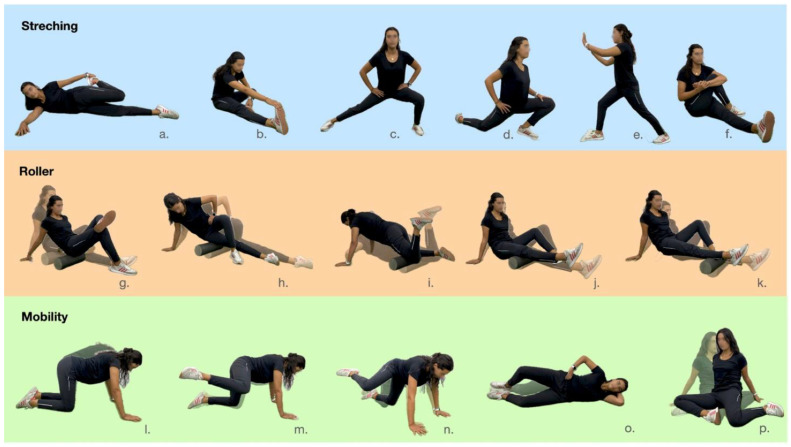
Exercises performed in each recovery strategy, presented in the order of execution. (**a**–**f**) passive stretching exercises focused on quadriceps, hamstrings, adductors, hip flexors, calf muscles, and gluteus; (**g**–**k**) foam rolling exercises applying self-massage over the anterior, posterior, and lateral regions of the thighs and legs (initial and final position); (**l**–**p**) lumbopelvic mobility exercises (initial and final position).

Ten min after the end of each match, participants who played for more than fifty min were randomly assigned to one of three different recovery protocols, that were carried out at the sports facility itself: (A) The stretching recovery strategy (stretching) that consisted of six passive stretching exercises focused on the thigh and leg muscles. Players performed three sets of 20 s in each exercise for each limb, always in the same order: quadriceps, hamstrings, adductors, hip flexors, calf muscles, and gluteus (Figure 1). This protocol was used as a control. (B) The foam rolling protocol (roller) used a foam roller (36″ length and 6.5″ diameter) to apply self-massage over the anterior, posterior, and lateral regions of the thighs and legs (i.e., five areas) in both lower limbs (Figure 1). Three sets of 20 s were performed alternately in each region. Players started at the proximal region of the thighs and legs and rolled down towards the distal region. Participants were instructed to utilize their body mass over their thighs and legs with the help of their arms. The speed was controlled using a metronome (2 s per pass). (C) The lumbopelvic mobility (mobility) recovery protocol consisted of five exercises focused on the lumbopelvic complex. The players performed three sets of 20 s in each exercise for each limb alternating between exercises, always in the same order (Figure 1). Speed was controlled using a metronome. The recovery strategies were developed in a quiet and temperature-controlled environment for approximately 10 min.

#### 2.3.2. Testing Procedures

Although all procedures were used as part of the normal training plan, one week before the start of this study, all participants were familiarized with the testing procedures and recovery strategies. Participants refrained from physiotherapy (e.g., massage, electrotherapy, ultrasound, heat treatment, cryotherapy, and hydrotherapy) before and after any measurement to avoid interference with the results. All the testing procedures were performed at the sports facility itself, under the same conditions and conducted by the same test leaders to ensure reliability and consistency in the results.

CMJA was tested 10 min after the official match (pre), immediately after the recovery protocol (post), and 5 h later (post 5). The participants’ jump heights were measured using a contact platform (Ergojump Bosco System, Byomedic, Barcelona, Spain). The participants started in a standing straight position and were instructed to jump as high as possible with their free hands. Jump height was recorded to the nearest 0.1 cm with a partial reliability (ICC) of 0.98 (95% CI: 0.95–0.99). Two trials, with a 30 s recovery, were allowed, and the best result was included in the data analysis.

Twenty-four h after the match, at the sport facilities and individually, players were instructed to complete an adapted version of a customized digital perceived wellness questionnaire [32] with which they were familiar, as it was used as part of the normal training plan. The questionnaire was designed to be brief, precise, and based on the components of self-perceived tools to assess players’ wellness [32]. Each player was asked to provide details about the following well-being and recovery variables: sleep quality and duration, fatigue, muscle soreness, and stress. All parameters were measured using a Likert scale ranging from 1 to 4, where 1 represented “very, very low” (fatigue, muscle soreness, and stress) or “poor” (sleep quality and duration), and 4 represented “very, very high” (fatigue, muscle soreness, and stress) or “optimal” (sleep quality and duration).

### 2.4. Statistical Analysis

All statistical analyses were performed using Jamovi software package (Jamovi Project, v.1.6.23.0; downloaded from https://www.jamovi.org, accessed on 1 November 2024). Normality was checked using the Shapiro–Wilk normality test. Then, a repeated-measures linear mixed model fitted with a restricted maximum likelihood method and unstructured covariates with Tukey post hoc adjustment was used to compare outcomes between the time (pre, post, and post 5 h) and recovery protocols (stretching, roller, and mobility). Tukey’s post-hoc adjustment was used. The main outcomes used in the statistical analyses were CMJA height (cm) and wellness perception (0–4 scale). The effect size (ES) was calculated for interactions between groups using Cohen’s guidelines. The threshold values for ES were >0.2 (small), >0.6 (large), and >2.0 (very large) [33]. The level of significance for all tests was set at *α* = 0.05. The mean, standard error (SE), and t values are reported for all statistical analyses.

## 3. Results

**Figure 2:**
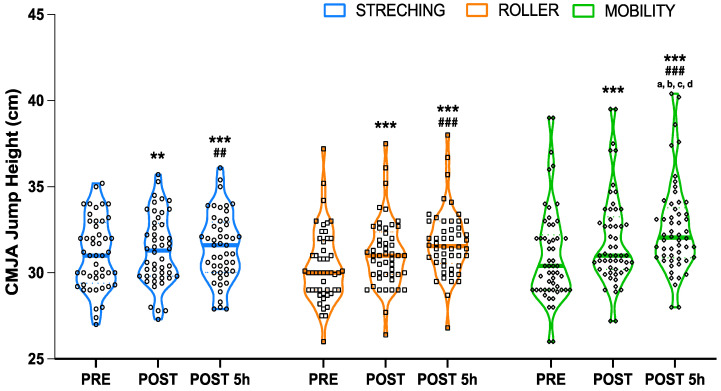
Mean (wider horizontal line) and SD (dashed horizontal lines) of vertical jump height before (pre), immediately after (post), and 5 h (post 5) post-recovery strategy for the three interventions (stretching, blue; foam roller, orange; and mobility, green), including individual responses (plots). * Significant (*p* < 0.05) intragroup differences compared to the pre-measurement, where ** means *p* < 0.01 and *** means *p* < 0.001, ^#^ Significant (*p* < 0.05) intragroup differences compared to the post-measurement, where ^##^ means *p* < 0.01 and ^###^ means *p* < 0.001. ^a^, Significant (*p* < 0.05) intergroup differences compared to the pre-measurement in the stretching condition. ^b^, Significant (*p* < 0.05) intergroup differences compared to the post-measurement in the stretching condition. ^c^, Significant (*p* < 0.05) intergroup differences compared to the pre-measurement in the mobility condition. ^d^, Significant (*p* < 0.05) intergroup differences compared to the post-measurement in the mobility condition.Regarding wellness perception, the statistical analysis revealed significant effects (*p* < 0.001, F = 30.3). Thus, post-hoc analysis showed significant increases in wellness perception after both roller (mean [*p*, t]: 0.75 [*p* < 0.001, t = 6.28]) and mobility recovery protocols (0.90 [*p* = 0.012, t = 7.65]) compared with stretching. Although the effect size with respect to the stretching protocol was higher after mobility (ES = 0.94 vs. ES = 0.88), no between-group differences were observed between the mobility and roller protocols.

Regarding CMJA vertical jump height, significant effects (*p* < 0.001) were observed for time (F = 56.6) and time*protocol (F = 59.6). Thus, as shown in Figure 2, post-hoc analysis showed significant enhancements in vertical jump height after all recovery protocols immediately after intervention (mean [SE, *p*, t, ES]; stretching: 0.22 [SE = 0.05, *p* = 0.010, t = 3.67, ES = 0.63]; roller: 0.75 [SE = 0.06, *p* < 0.001, t = 12.72, ES = 1.34]; mobility: 0.89 [SE = 0.06, *p* < 0.001, t = 15.76, ES = 2.96]) and 5 h post-intervention (stretching: 0.40 [SE = 0.07, *p* < 0.001, t = 5.53, ES = 0.85]; roller: 1.38 [SE = 0.07, *p* < 0.001, t = 19.70, ES = 2.23]; mobility: 1.58 [SE = 0.07, *p* < 0.001, t = 23.35, ES = 4.17]) compared to pre-intervention measurements. Similarly, when CMJA vertical jump height scores were compared between post-intervention and 5 h post intervention measurements, higher values (*p* < 0.01) were observed at post 5 h for all protocols (stretching: 0.18 [SE = 0.04, *p* = 0.002, t = 4.19, ES = 0.48]; roller: 0.63 [SE = 0.04, *p* < 0.001, t = 15.43, ES = 2.57]; and mobility: 0.68 [SE = 0.04, *p* < 0.001, t = 17.29, ES = 2.71]). Additionally, significant differences were observed between the groups. The mobility protocol showed higher vertical jump height results 5 h after intervention when compared to stretching (pre: 1.48 [SE = 0.45, *p* = 0.036, t = 3.26]; post: 1.26 [SE = 0.45, *p* = 0.018, t = 3.82]) and roller (pre: 1.48 [SE = 0.45, *p* = 0.036, t = 3.26]; post: 1.46 [SE = 0.44, *p* = 0.033, t = 3.29]). Moreover, the mobility protocol showed higher CMJA values immediately after the recovery protocol than those before the intervention (1.52 [SE = 0.45, *p* = 0.027, t = 3.35]). Finally, between-group differences were also observed 5 h post-intervention between the mobility and stretching protocols, showing higher vertical jump height values after the mobility protocol (1.09 [SE = 0.44, *p* = 0.040, t = 2.56]). No other differences were observed between the groups at any time point.

## 4. Discussion

Given the scarcity of scientific evidence concerning effective training interventions to expedite post-match recovery, this study aimed to evaluate the efficacy of active recovery strategies in young female football players during competitive tournaments with limited recovery times (i.e., 24–48 h). The primary findings indicate that all recovery protocols significantly enhanced CMJA vertical jump height both immediately after the intervention and 5 h post-intervention compared to pre-intervention levels. Notably, the lumbopelvic mobility protocol demonstrated the greatest improvements, significantly outperforming both the stretching and foam rolling protocols in enhancing vertical jump height. Furthermore, significant increases in wellness perception were observed following both the foam rolling and mobility protocols, with the mobility protocol demonstrating a slightly higher effect size than the stretching protocol. These results underscore the superior efficacy of the lumbopelvic mobility protocol in improving neuromuscular function and wellness perception in young female football players.

Recovery is a multifaceted process influenced by time and various internal and external factors, depending on stress reduction or disruption [19,34]. It impacts neuromuscular efficiency detectable through decreased vertical jump performance [35,36]. In team sports like football, congested schedules highlight the need for recovery strategies to restore autonomic nervous system activity, reduce severity and duration of fatigue, diminish its perception and optimize neuromuscular performance [12,13,29,30]. Therefore, recovery is essential for improving performance and preventing injuries, and it can be classified into passive and active strategies [28]. As explained previously, passive recovery techniques aim to reduce muscle soreness and inflammation, while promoting relaxation and recuperation without further physical exertion [14,15,16,31]. On the other hand, active recovery involves low-intensity exercises designed to maintain blood flow and accelerate the removal of metabolic waste products [22,23,24,28]. In football, active recovery practices are often employed post-match to expedite muscle repair and reduce fatigue [17]. Research suggests that active recovery can outperform passive methods in sports that requiring high neuromuscular function and repeated high-intensity efforts, like football [29,30,37].

Although our results contribute to the growing body of evidence demonstrating that active recovery protocols can significantly enhance recovery outcomes in young female football players, the effectiveness of these protocols has not been demonstrated previously. In fact, present results are in contrast to those of Andersson et al. [6], who found no significant differences in neuromuscular recovery (e.g., countermovement jump height, running speed, peak torque values in knee flexion and extension) or muscle damage biomarkers (creatine kinase, uric acid, and urea) between active recovery protocols (i.e., cycling at 60% HRmax and low-intensity resistance training at 50% of the 1-RM) and passive recovery protocols (i.e., resting). Similarly, Andersson et al. [23] investigated plasma antioxidants and oxidative stress markers in elite female football players after two 90 min games. Surprisingly, active recovery (low-intensity cycling and resistance training) did not significantly affect oxidative stress markers or antioxidants. However, endogenous antioxidants increased immediately after both games, and dietary antioxidants showed rapid and persistent changes after the first game (increased tocopherols and ascorbic acid and decreased polyphenols). Similarly, these authors also noted that low-intensity exercise did not influence pro-inflammatory cytokine responses (e.g., TNF-α, IFN-γ, IL-12) or anti-inflammatory cytokines (e.g., IL-4, IL-5, IL-6, IL-10, INF-α) [38]. These findings suggest that low-to-moderate intensity aerobic exercise combined with low-intensity strength exercises may not provide substantial improvements in muscle recovery compared to resting. On the other hand, to date, the effect of other strategies, such as foam rolling or mobility exercises, on perceptual and functional recovery is unknown. However, our study indicates that both foam rolling and mobility exercises might offer distinct benefits not captured in these earlier studies, particularly in terms of immediate and short-term recovery outcomes.

Present results are in line with the previous literature showing that stretching and foam rolling are effective strategies owing to their benefits for muscle function [39,40], flexibility, and psychological relaxation [41,42]. Moreover, they can help alleviate muscle soreness [43] and restore joint mobility after a match [44]. Additionally, by applying pressure to the muscles, foam rolling helps break up adhesions and scar tissue, reduce tightness in the connective tissue surrounding the muscles, alleviate pain and discomfort, and further increase flexibility and range of motion [20]. These benefits contribute to maintaining proper movement patterns, reducing the risk of injury, and improving performance in subsequent workouts [43,45]. Additionally, foam rolling allows for a more personalized approach as individuals can adjust the pressure applied to specific muscles or areas of tightness more precisely than stretching [39]. It also provides immediate pain relief by stimulating pressure receptors in muscles and fascia [20], thereby reducing pain perception and promoting lymphatic fluid flow to reduce muscle inflammation and swelling [20,46,47]. This could lead to a better perception of wellness than stretching.

However, our results revealed that lumbopelvic mobility exercises offer superior benefits compared with foam rolling or stretching. These exercises engage multiple muscle groups simultaneously, providing a holistic approach to recovery by addressing mobility, stability, and neuromuscular control [48,49] while also restoring proper posture and movement patterns, particularly in the lower back and pelvis, which are often stressed during high-intensity activities [48]. They improve overall mechanics, increase flexibility and movement range, alleviate stiffness, and enhance coordination between the nervous system and muscles [50]. By targeting these key areas, lumbopelvic mobility exercises promote both physical recovery and prevent potential compensatory movements that could lead to further injury [50]. In addition, these exercises contribute to relaxation of the central nervous system and muscles, fostering overall recovery and mental well-being after intense physical activity.

Although this study provides valuable insights into the efficacy of active recovery strategies in young female football players, it is not without its limitations. One primary limitation is the relatively small sample size, which may have affected the generalizability of the findings. Additionally, this study’s focus on a specific age group and sex limits the applicability of the results to other populations such as male athletes or players of different age categories. Another constraint is the short-term nature of the interventions and measurements. However, the long-term effects of these recovery strategies remain unknown. Furthermore, this study did not include physiological variables, such as blood concentration of creatine kinase (CK) or other pro-inflammatory cytokine responses or heart rate variability (HRV), which are important indicators of muscle damage and autonomic nervous system activity, respectively. The exclusion of these variables limits our understanding of the physiological mechanisms that underlie recovery. Future research should include larger and more diverse cohorts to enhance the generalizability of these findings. Longitudinal studies examining the long-term effects of different recovery protocols have provided deeper insights into their sustained efficacy. Additionally, future investigations should consider controlling for or measuring additional variables such as nutrition, sleep, CK and pro-inflammatory cytokine levels, and HRV to better understand their interactions with recovery strategies. Exploring the molecular and physiological mechanisms underlying the benefits of recovery protocols could offer valuable information for optimizing recovery practices in sports.

## 5. Conclusions

In conclusion, this study demonstrated that active recovery strategies significantly enhanced neuromuscular function and wellness perception in young female football players during competitive tournaments with limited recovery times. Among the recovery protocols examined, lumbopelvic mobility exercises showed superior efficacy in improving vertical jump height and wellness perception, outperforming both the stretching and foam rolling techniques. These findings highlight the importance of incorporating structured active recovery interventions to optimize the performance and recovery of female football athletes. For example, a concrete alternative could be to include a stable protocol of 3–7 exercises, performed immediately after the match, that combines lumbopelvic mobility with foam roller, aiming to facilitate recovery from both a physical and psychological perspective. In any case, although all protocols proved beneficial, the pronounced effects of lumbopelvic mobility exercises suggest that they should be prioritized in post-match recovery routines.

## Data Availability

The data supporting the findings of this study are available from the corresponding author upon reasonable request or can be accessed at https://doi.org/10.5281/zenodo.14167811. (accessed on 1 January 2025).
